# Osteosarcopenia predicts greater risk of functional disability than sarcopenia: a longitudinal analysis of FraDySMex cohort study

**DOI:** 10.1016/j.jnha.2024.100368

**Published:** 2024-09-21

**Authors:** Oscar Rosas-Carrasco, Betty Manrique-Espinoza, Juan Carlos López-Alvarenga, Beatriz Mena-Montes, Isabel Omaña-Guzmán

**Affiliations:** a Geriatric Assessment Center, Health Department, Iberoamerican University, Mexico City, Mexico; b National Institute of Public Health, Morelos, Mexico; c Population Health & Biostatistics, University of Texas Rio Grande Valley, Texas, USA; d National Institute of Geriatrics, Mexico City, Mexico; e Pediatric Obesity Clinic and Wellness Unit, Hospital General de México “Dr. Eduardo Liceaga”, Mexico City, Mexico

**Keywords:** Functional disability, Sarcopenia, Osteoporosis, Osteosarcopenia, Older adults, Body composition, Cohort study.

## Abstract

**Objectives:**

Aging involves significant changes in body composition, marked by declines in muscle mass and bone mineral density alongside an increase in fat mass. Sarcopenia is characterized by low strength and muscle mass, and osteosarcopenia is the coexistence of sarcopenia and osteopenia/osteoporosis. Physiologically, there is a crosstalk between muscle and bone tissues mediated by several pathways. Both, sarcopenia and osteosarcopenia, have been related with adverse outcomes such as functional disability. However, there is a lack of longitudinal studies. Therefore, this study aimed to assess whether sarcopenia and osteosarcopenia phenotypes increased the risk of functional disability in a longitudinal cohort of community-dwelling adults.

**Design:**

This study constitutes a secondary longitudinal analysis of data derived from the prospective cohort FraDySMex (Frailty, Dynapenia, and Sarcopenia in Mexican adults).

**Setting and participants:**

FraDySMex is conducted in community-dwelling adults aged 50 years or older living in Mexico City. Data from 2014 to 2015 was considered as baseline evaluation, and the 2019 wave was the follow-up evaluation. Individuals with complete baseline and follow-up evaluations were included in the analysis.

**Measurements:**

Sarcopenia diagnosis adhered to the FNIH criteria, while osteopenia/osteoporosis classification followed WHO guidelines. Osteosarcopenia was defined as the concurrent presence of sarcopenia and osteopenia/osteoporosis. Functional disability was identified by the Lawton Instrumental Activities of Daily Living (IADL) Scale. Adjusted mixed-effects logistic regression models were estimated to evaluate the effect of body composition phenotype on the risk of functional disability.

**Results:**

The final sample included 320 adults with complete longitudinal data. The majority of were women (83.4%) and had 7–12 years of education (48.4%). At the baseline evaluation, 50.9% aged 50–70. The osteosarcopenia phenotype was associated with a higher risk of functional disability (OR: 2.17, p = 0.042) compared with the no osteopenia/sarcopenia group. Conversely, sarcopenia (OR: 1.50, p = 0.448) and osteopenia/osteoporosis (OR: 1.50, p = 0.185) phenotypes were not associated with functional disability.

**Conclusions:**

Our study underscores that osteosarcopenia significantly increased the risk of functional disability, particularly in terms of Instrumental Activities of Daily Living (IADL). These results emphasize the importance of screening for sarcopenia, osteopenia/osteoporosis, and osteosarcopenia across various clinical settings. Early detection and intervention hold promise for averting functional disability and mitigating associated adverse outcomes in adults.

## 1 Introduction

Aging involves changes in body composition characterized by a decrease in muscle mass and bone mineral density (BMD) and an increase in fat mass (FM) [1,2]. Consequently, several phenotypes involving alterations in these tissues, such as sarcopenia, osteopenia/osteoporosis, osteosarcopenia, sarcopenic obesity and osteosarcopenic obesity, have been described in older adults.

Sarcopenia is a musculoskeletal condition characterized by a low muscle quantity and quality that affects muscle function [3]. It is associated with adverse outcomes such as falls [4], low quality of life [5], functional decline, and mortality, among others [4]. It has been estimated that this pathological condition affects from 10% to 27% of older adults worldwide [6]. The pathophysiology of sarcopenia involved neurodegenerative processes, cellular senescence, oxidative stress, mitochondrial dysfunction, low-grade inflammation, hormonal changes, and reduction in satellite cells' number and regeneration capacity [7,8]. Otherwise, osteopenia/osteoporosis has BMD reduction, affecting the bone microarchitecture increasing risk of fractures [9].

The coexistence of sarcopenia and osteopenia/osteoporosis in the same individual has recently been named osteosarcopenia [9]. Recent meta-analyses have estimated a pooled prevalence of osteosarcopenia at 12.8% [10], 7.2% [10] and 20.7% [11], along with its association with an increased risk of fractures, falls, and mortality [11]. Muscle and bone constitute two intricately connected tissues essential for mobility that are physiologically correlated at biochemical, cellular, and endocrine levels. The crosstalk between these two tissues is not completely understood and continues to be studied. Several biochemical pathways have been implicated in the pathophysiology of osteosarcopenia from the point of view of geroscience [12]. The muscle provides mechanical stimuli (contraction) on the bone, exerting the tension necessary for optimal bone metabolism [13]. Increasing muscle mass stimulates the periosteum and bone growth, reducing the risk of fractures [14].

From a genetic point of view, a study in twins revealed that lean mass and BMD have more genes in common than fat mass and BMD [15]. At the level of muscle-bone intercommunication, it has been shown that some myokines (insulin-like growth factor 1 (IGF1), fibroblast growth factor, follistatin, osteonectin, osteoglycin, irisin, interleukin-15 (IL-15), myostatin (GDF8) and interleukin-6 (IL-6)) act positively or negatively on bone, while osteokines (osteocalcin, connexin 43 and sclerostin) play a role in muscle [[16], [17], [18]]. This bidirectional secretion is important for healthy muscle and bone metabolism [17]. Indeed, muscle and bone have their origin in a common precursor, mesenchymal stem cells (MSCs) [19].

There is another crucial pathway wherein myogenic progenitors (MyoD lineage cells) migrate to open fractures. In this site, MyoD cells initially differentiated into a chondrogenic phenotype, followed by an osteoblastic phenotype [20,21]. Recently, it has been described that skeletal muscle satellite cells have a role in osteoporotic fracture repair mediated by β-catenin signaling [22]. This process highlights the significant crosstalk between muscle and bone, essential for maintaining homeostasis.

Likewise, aging entails a decrease in functional ability. Functional ability is the primary goal of healthy aging and, generally, is evaluated in terms of basic activities of daily living (BADLs) or instrumental activities of daily living (IADLs) [23]. IADLs comprises activities related to finances, transport, home care, shopping, and medication take, among others. Disability in terms of BADLs and IADLs has been associated with adverse consequences such as low quality life [24] and morbidity [25]. Considering that the older population is increasing worldwide, it is urgent to identify the factors affecting functional abilities to avoid healthy aging. Sarcopenia and osteosarcopenia have been associated with functional disability [[26], [27], [28]]. However, there is a lack of longitudinal studies about these conditions that corroborate the temporality and magnitude of the risk to functional disability (basic and instrumental activities) along with other risk factors already demonstrated previously. Therefore, the objective of this study was to evaluate if sarcopenia and osteosarcopenia phenotypes increase the risk of functional disability in a cohort study of community-dwelling adults.

## 2 Material and methods

### 2.1 Design and population study

This is a secondary longitudinal analysis of the prospective cohort FraDySMex (Frailty, Dynapenia and Sarcopenia in Mexican adults) that is conducted in community-dwelling adults aged 50 years or older living in two municipalities of Mexico City. FraDySMex included individuals with the following characteristics: (1) capable of moving with or without assistive devices, (2) able to answer the study questionnaire for themselves or with the help of a caregiver, (3) a total score of Mini-Mental State Examination (MMSE) ≤10 points. The exclusion criteria were institutionalized individuals, those with decreased alertness, and the presence of any acute or chronic condition that, according to the opinion of the medical staff, could affect the individual’s ability to answer proposed questionnaires and complete the objective evaluation. The first wave of the cohort was conducted in 2014 (n = 339), the second in 2015 (n = 491) and the third in 2019 (n = 852). In this study data from 2014 to 2015 was considered as baseline evaluation. Individuals with complete measurements in 2014 and those who had their first complete evaluation in 2015 were included (total sample: n = 320) to the analysis. The 2019 wave was the follow-up evaluation. We included individuals with two assessments (baseline and follow-up).

The participants were evaluated by a multidisciplinary team at the Functional Evaluation Research Laboratory at the National Geriatric Institute (2014–2015 and 2019 waves) and the Geriatric Assessment Center at the Iberoamerican University (2019 wave).

This research was conducted following the Declaration of Helsinki. All participants signed informed consent letter. The study was approved by the Ethics Committee of the Angeles Mocel General Hospital (CONBIOETICA-09-cei-013- 20170517/2019) and registered by the National Geriatrics Institute (DI-PI-002/2014).

### 2.2 Sarcopenia, osteopenia/osteoporosis and osteosarcopenia

Body composition was evaluated using dual-energy X-ray absorptiometry (DXA) (Hologic Discovery-WI; Hologic, Bedford, MA). Grip strength was measured with a hydraulic hand dynamometer (Jamar, Duluth, MN). Three measurements were taken from each hand for each participant, and the highest result from the dominant hand was considered the final value.

We considered the criteria proposed by the Foundation for the National Institutes of Health (FNIH) Biomarkers Consortium Sarcopenia Project consensus [3] for the diagnosis of sarcopenia which included Hispanic population: low appendicular lean mass (ALM) adjusted for body mass index (BMI) (ALM_BMI_) and low handgrip strength. The cutoff points to sarcopenia diagnosis were: for low ALM_BMI_ was <0.789 for men and <0.512 for women, and low handgrip strength was <26 kg for men and <20 for woman [29].

For osteopenia/osteoporosis, the T-score of right and left hip BMD was used (the higher value was considered). If these values were missing (five percent of the data), we used the T-score of the whole BMD, the correlation between these two measurements was good (r = 0.64). Osteopenia/osteoporosis was defined according to WHO cutoff values as a T-score ≤1.0 [30].

Osteosarcopenia was considered as the coexistence of sarcopenia and osteopenia/osteoporosis.

### 2.3 Functional disability

Functional disability was assessed with the Lawton Instrumental Activities of Daily Living Scale. This scale comprises eight activities: (1) Ability to use telephone; (2) Shopping; (3) Food preparation; (4) Housekeeping; (5) Laundry; (6) Mode of transportation; (7) Responsibility for own medications and 8) Ability to handle finances [31]. Each activity has 3–5 possible responses with a score of zero or one; The maximum total score is 8. It was considered as disability for IADL if the total score was ≤7 (at least one task could not be performed).

### 2.4 Sociodemographic and clinical variables

Information about age, sex, education, and marital status was obtained from the sociodemographic questionnaire applied in each evaluation wave. Obesity was defined as a fat percentage >40 for women and >30 for men [32]. The presence of comorbidity was assessed using the Charlson Index [33]. Low comorbidity was considered if the total score was <3, and high comorbidity if it was ≥3. Polypharmacy was defined as the use of five or more medications per day [34]. The MMSE [35] was used to evaluate cognitive function. In individuals with five or more years of study, a total score ≤23 was considered as a probable cognitive impairment; for those with one to four years of study, the score was ≤19, whereas a total score ≤16 was considered for individuals with less than one year of study. Undernutrition or undernutrition risk was evaluated with the Mini Nutritional Assessment (MNA) [36]. A total score ≥17 was defined as undernutrition/ undernutrition risk. Participants were categorized as sedentary if they did not engage in any exercise or participated in physical activity for less than two hours per week. Regular physical activity was defined as performing 2–4 hours of activity per week.

### 2.5 Statistical analysis

To describe the study population, means and standard deviations (SD) were estimated for quantitative variables and for categorical variables, frequencies and proportions were calculated and differences were evaluated with chi-squared tests.

**Fig. 1:**
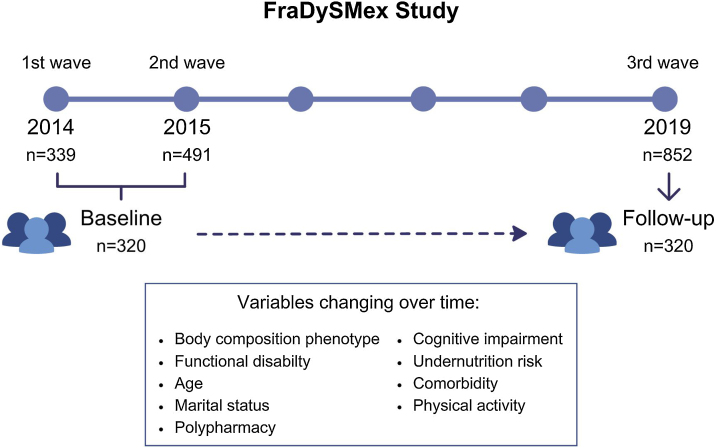
FraDySMex study design.

Given the longitudinal design of our study and the evolving nature over time of both the outcome variable (functional disability) and the primary independent variable (sarcopenia, osteopenia/osteoporosis and osteosarcopenia), we used mixed-effects logistic regression models (MELRM) to assess their association. MELRM consider variability both between and within subjects, enabling us to model changes in both dependent and independent variables (Fig. 1).

Adjusted MELRM were performed to evaluate the association between functional disability and sarcopenia and osteosarcopenia phenotypes. The final model was adjusted for age, sex, marital status, obesity, comorbidity, polypharmacy, undernutrition risk, cognitive impairment, and physical activity. To investigate whether the presence of obesity influenced the association between body composition phenotypes and functional disability, we introduced an interaction term between these variables into the model for analysis. A p-value <0.05 was considered statistically significant. All analyses were performed using STATA 15.0.

## 3 Results

### 3.1 Baseline sociodemographic and follow-up clinical characteristics

A total of 320 participants with baseline and follow-up evaluations were included (Fig. 1). In Table 1 are shown the clinical and sociographic characteristics of the participants in the baseline and follow-up evaluations. At the baseline evaluation, almost half of the studied sample were aged 50–70 (50.9%), the majority were women (83.4%), had 7–12 years of education (48.4%), and 42.0% were married or living in consensual union. Obesity was presented in 60.3% of the participants. Only sarcopenia affected to 10.6% of the participants, osteopenia/osteoporosis to 42.5% and osteosarcopenia to 27.5%. Functional disability was present in 16.6% of the participants. Otherwise, in follow-up evaluation, the osteosarcopenia slightly increased to 29.7%, sarcopenia to 10.9% and osteopenia/osteoporosis decreased to 37.8%. The proportion of functional disability increased 9.4 percentual points. The proportion of participants with high comorbidity, undernutrition/ undernutrition risk, cognitive impairment and a sedentary life also increased.

**Table 1:** Baseline and follow-up characteristics of participants.

Characteristics	Baseline	Follow-up
**Body composition phenotypes**	**n (%)**	**n (%)**
No osteopenia/sarcopenia	75 (23.4)	84 (26.3)
Sarcopenia	21 (6.6)	20 (6.3)
Osteopenia/osteporosis	176 (55.0)	149 (46.6)
Osteosarcopenia	48 (15.0)	67 (20.9)
**Functional disability by Lawton’s scale**		
Without functional disability (≥8 points)	263 (82.2)	231 (72.2)
Functional disability (≤7 points)	57 (17.8)	89 (27.8)
**Obesity**		
No	127 (39.7)	143 (44.7)
Yes	193 (60.3)	177 (55.3)
**Age**		
50–70 years	163 (50.9)	114 (35.6)
≥70 years	157 (49.1)	206 (64.4)
**Sex**		
Women	267 (83.4)	267 (83.4)
Men	53 (16.6)	53 (16.6)
**Education (years)**		
≥13	79 (24.7)	79 (24.7)
7 a 12	155 (48.4)	155 (48.4)
0 a 6	86 (26.9)	86 (26.9)
**Marital status**		
Married/consensual union	134 (42.0)	120 (37.5)
Single, separated/divorced	80 (25.1)	87 (27.2)
Widower	105 (32.9)	113 (35.3)
**Charlson Index**		
Low comorbidity (≤2 points)	259 (80.9)	246 (76.8)
High comorbidity (≥3 points)	61 (19.1)	74 (23.2)
**Polypharmacy**		
No (≤4 medications)	188 (59.1)	186 (58.2)
Yes (≥5 medications)	130 (40.9)	134 (41.8)
**Undernutrition risk by MNA**		
Without risk (≥23.5 points)	241 (78.8)	197 (62.9)
Undernutrition/undernutrition risk (≤23 points)	65 (21.2)	116 (37.1)
**Cognitive impairment by MMSE**		
No	290 (90.6)	286 (89.4)
Yes	30 (9.4)	34 (10.6)
**Physical activity**		
Sedentary/low activity	232 (73.7)	243 (78.1)
Moderate/vigorous	83 (26.3)	68 (21.9)

Abbreviations: Abbreviations: MNA, Mini Nutritional Assessment; MMSE, Mini Mental State Examination.

Cognitive impairment by MMSE: If total score ≤23 and ≥ 5 years of study; total score ≤19 and 1–4 years of study; total score ≤16 and ≤1 years of study.

The most affected IADL between participants with osteosarcopenia in the baseline and follow-up evaluations were 'Mode of Transportation', 'Shopping' and 'Food Preparation' (Table 2).

**Table 2:** IADL affected in osteosarcopenia group in baseline and follow-up evaluations according Lawton’s Scale.

Activities	Baseline (n = 48)		Follow-up (n = 67)	
	n	%	n	%
Shopping	7	14.6	24	35.8
Mode of transportation	7	14.6	20	29.9
Food preparation	1	2.1	17	25.4
Laundry	3	6.3	12	17.9
Responsibility for own medications	1	2.1	8	11.9
Housekepping	0	0	7	10.8
Ability to use telephone	1	2.1	3	4.5
Ability to handle finances	0	0	3	4.5

### 3.2 Association between body composition phenotypes and functional disability

The osteosarcopenia phenotype characterized by alterations in muscle and bone tissues was associated with a higher risk of functional disability (OR: 2.17, p = 0.042) compared with the no osteopenia/sarcopenia group. Sarcopenia (OR: 1.50, p = 0.448) and osteopenia/osteoporosis (OR: 1.50, p = 0.185) phenotypes were not associated with functional disability (Table 3).

**Table 3:** Risk of Functional Disability: Effect of Body Composition Phenotypes and Other Factors. A Mixed-Effects Logistic Regression Model Analysis.

	OR	p-value	95%CI
**Body composition phenotype**			
No osteopenia/sarcopenia	REF	–	–
Sarcopenia	1.50	0.448	0.53 - 4.29
Osteopenia/osteporosis	1.50	0.185	0.82 - 2.75
Osteosarcopenia	2.17	0.042	1.03 - 4.56
**Sex**			
Women	REF	–	–
Men	1.77	0.139	0.83 - 3.77
**Age**			
50–70 years	REF	–	–
≥ 70 years	4.14	<0.001	2.19 - 7.85
**Marital status**			
Married/consensual union	REF	–	–
Single, separated/divorced	1.04	0.903	0.54 - 2.00
Widower	1.37	0.325	0.73 - 2.56
**Obesity**			
No	REF	–	–
Yes	1.25	0.332	0.77 - 2.05
**Charlson Index**			
Low comorbidity (≤ 2 points)	REF	–	–
High comorbidity (≥ 3 points)	1.19	0.526	0.70 - 2.02
**Polypharmacy**			
No (≤ 4 medications)	REF	–	–
Yes (≥ 5 medications)	1.62	0.071	0.96 - 2.73
**Undernutrition risk by MNA**			
Without risk (≥ 23.5 points)	REF	–	–
Undernutrition/undernutrition risk (≤ 23 points)	2.00	0.005	1.23 - 3.26
**Cognitive impairment by MMSE**			
No	REF	–	–
Yes	4.85	<0.001	2.33 - 10.11
**Physical activity**			
Sedentary/low	REF	–	–
Moderate/vigorous	0.89	0.703	0.49 - 1.61
**Time**	1.33	0.213	0.84 - 2.12

Abbreviations: MNA, Mini Nutritional Assessment; MMSE, Mini Mental State Examination.

Cognitive impairment by MMSE: If total score ≤23 and ≥5 years of study; total score ≤19 and 1–4 years of study; total score ≤16 and ≤1 years of study.

Final model adjusted by sex, age, marital status, obesity, comorbidity, undernutrition risk, cognitive impairment, and physical activity.

We tested an interaction between body composition phenotypes and obesity in the model, which was not significant. Therefore, obesity was used just as covariable in the final model.

Other variables associated with an increased the risk of functional disability were age ≥70 (OR: 4.14, p < 0.001), undernutrition risk (OR: 2.00, p = 0.005) and cognitive impairment (OR: 4.85, p < 0.001).

## 4 Discussion

Osteosarcopenia increased the risk of functional disability in community-dwelling adults aged 50 and over. Otherwise, sarcopenia and osteopenia/osteoporosis, were not associated with functional disability. Furthermore, obesity did not increase the risk of this outcome in our population.

The predictive capacity of different body composition phenotypes for functional disability has been underexplored. Additionally, studies have not distinguished adequately which specific tissues had more relevance to predict this outcome, and there is a lack of longitudinal analysis.

Opposite to the evidence [26], we did not find that sarcopenia increased the risk of functional disability. However other studies have not differentiated between sarcopenia and osteosarcopenia and as we presented, muscle and bone alterations had an independent effect on functional disability. Regarding osteosarcopenia there is a bit more evidence. A cross-sectional study conducted with the same population of FraDySMex cohort [27] found a positive association with functional disability. Nevertheless, the osteopenia/osteoporosis phenotype was not considered in the analysis and no distinction was made between BADL and IADL. In Japanese older adults [37] also it has found a positive correlation with functional disability, but neither was a distinction made between BADL and IADL.

An important domain of functional abilities is the capacity for physical movement related with the sensory-motor functioning of the body [38] and the musculoskeletal mechano-transduction [39], which are affected by the alterations in muscle and bone tissues that characterize the osteosarcopenia [39]. The pathophysiology of osteosarcopenia involves significant disruptions in the muscle-bone crosstalk, leading to impaired functioning of both tissues [40]. A key aspect of this condition is a dysregulation characterized by decreased levels of myokines and osteokines with positive regulatory functions, and increased levels of those with negative regulatory roles. For instance, low levels of irisin, a myokine associated with muscle hypertrophy [41], which also promotes bone formation and reduce osteoclast activity [42]. Conversely, elevated levels of myostatin, a myokine linked to muscle atrophy and decreased muscle mass [43,44], exacerbate these conditions. Myostatin additionally acts as a positive modulator of osteoclast differentiation and exerts anti-osteogenic effects, impacting BMD [41,44]. Other myokines, such as fibroblast growth factor 21 (FGF-21), interleukin 15 (IL-15), brain-derived neurotrophic factor (BDNF), follistatin and IGF-1, which have positive regulatory effects on muscle and bone, are also dysregulated in osteosarcopenia [[42], [43], [44]]. Similarly, osteokines that exert both positive effects (e.g., osteocalcin) and negative effects (e.g., RANKL, SOST, and sclerostin) on bone and muscle are also dysregulated [42,44].

Moreover, osteosarcopenia is associated with a pro-inflammatory environment characterized by elevated levels of TNFα, IL-6, and C-reactive protein (CRP) [45]. This inflammatory state is further exacerbated by fat infiltration into myocytes, negatively impacting both muscle and bone tissues [[46], [47], [48]]. Additionally, muscle produces excessive levels of reactive oxygen species (ROS) leading to oxidative stress which in turn increases inflammation [49].

As a result of these alterations, muscle strength, muscle mass, and BMD decreased, which impairs mobility, gait, and balance, leading to a decline in an individual's functional capacity [50]. Consequently, the most affected IADL were those related with movement and strength such as transportation, shopping and food preparation.

Recent research has explored the muscle-brain axis, where myokines play a crucial role in regulating and mediating cognitive functions and higher-order processes, such as learning, memory, and coordination [51]. Consequently, the dysregulation of myokines observed in osteosarcopenia may also impact IADL that require advanced cognitive and executive functions, including tasks like 'Ability to Use a Telephone', 'Responsibility for Own Medications', and 'Ability to Handle Finances'.

Otherwise, studies have been documented that sarcopenic obesity increase the risk of functional disability [52], but alterations in BMD to identify the phenotypes were not been considered. In our population, obesity did not play a significant role in predicting disability. Although it has been described that adipose tissue can worsen the musculoskeletal alterations present in osteosarcopenia through its infiltration into the muscle [9], we found that excessive body fat not affect the capacity to perform IADL. These results support the idea that the simultaneous presence of muscle and bone alterations has a more significant impact on functional disability than combined alterations in adipose tissue and muscle or in a single tissue such as bone, muscle, or fat. The above suggest that the critical factors influencing functional ability in terms of IADL are the altered mechanisms in the muscle-bone physiology. Studies focusing on these altered mechanisms are needed. However, the presence of obesity does not reflect the fat infiltration into the myocytes and this mechanism deserves to be explored.

Additional characteristics that increased the risk of functional disability included age ≥70, undernutrition or risk of undernutrition and symptoms indicating cognitive impairment. These variables are well-documented risk factors for functional disability, and our findings align with those reported in other populations [[53], [54], [55]].

It is crucial to distinguish between BADL and IADL when assessing functional disability, as these dimensions reflect different degrees of individuals' functional capacity. The functional capacity associated with IADLs could be affected earlier than that associated with BADLs, therefore, identifying this could allow timely intervention aimed at improving associated modifiable factors, including alterations in body composition.

Some limitations of this study must be considered. First, we did not have a probabilistic sample; therefore, the findings cannot be extrapolated to other populations. Furthermore, we could not include in the adjusted model the effect of variables such as energy and macronutrient intakes that are associated with the studied variables. Nevertheless, the study has important strengths related to its prospective design and robust statistical analysis, which allow us to model changes in both dependent and independent variables in a continuous and categorical approach. Moreover, we have detailed sociodemographic and clinical data of the participants that included objective measurements performed with high-quality diagnosis tools.

## 5 Conclusions

In conclusion, osteosarcopenia increases the risk of functional disability in terms of IADL. Our findings highlight the importance of diagnosing sarcopenia, osteopenia/osteoporosis, and osteosarcopenia, as well as assessing functional capacities related to both IADL and BADL in individuals aged 50 years and older as part of comprehensive clinical evaluations. Additionally, nutritional status and cognitive function should be assessed. An exhaustive evaluation is critical for preventing and managing body composition alterations and functional disability through personalized multidisciplinary interventions. These interventions should prioritize nutritional strategies and physical exercise, which has been proven to enhance muscle mass, strength, and BMD in older adults [56] thereby enhancing physical capacity and improving IADL [57].

## Funding

This study was supported by the “Programa presupuestario con erogación para la igualdad entre mujeres y hombres de la Secretaría de Salud (2014–2015)”, and by the "Red colaborativa de la investigación traslacional para el envejecimiento saludable” (RECITES-2019) in the Instituto Nacional de Geriatría, Mexico City”.

## Ethical standards

This research was conducted following the Declaration of Helsinki and its later amendments. All participants signed informed consent letter prior to their inclusion in the study. The study was approved by the Ethics Committee of the Angeles Mocel General Hospital (CONBIOETICA-09-cei-013- 20170517/2019) and registered by the National Geriatrics Institute (DI-PI-002/2014).

## Conflict of interest

The authors declare no conflict interests.
